# Effects of Virtual Reality-Based Rehabilitation on Postural Balance in Older Adults: A Systematic Review

**DOI:** 10.3390/geriatrics11050143

**Published:** 2026-10-07

**Authors:** Barbara O. M. Neiva, Andressa Maysa S. Martins, Murilo Antônio R. Beze, Silvia Priscilla R. Oliveira, João Pedro R. Afonso, Rodrigo Antônio C. Andraus, Rodrigo F. Oliveira, Deise A. A. P. Oliveira, Tiago V. Fernandes, Daniel G. Marconi, Karla Cristina N. Carvalho, Claudia S. Oliveira, Carlos Hassel M. Silva, Orlando A. Guedes, Luciana P. Maia, Paolo Capodaglio, Miriã C. Oliveira, Luis Vicente F. Oliveira

**Affiliations:** 1 Graduate Program in Human Movement and Rehabilitation (PPGMHR), Evangelical University of Goiás (UNIEVANGELICA), Anápolis 75083-515, GO, Brazil; barbara.fisioterapia@hotmail.com (B.O.M.N.); joaopedro180599@gmail.com (J.P.R.A.); rodrigoandraus@gmail.com (R.A.C.A.); rodrigofranco65@gmail.com (R.F.O.); deisepyres@gmail.com (D.A.A.P.O.); medkarcri@yahoo.com.br (K.C.N.C.); csantos.neuro@gmail.com (C.S.O.); miriacandidaoliveira@gmail.com (M.C.O.); 2 Department of Physical Therapy, Evangelical University of Goiás (UniEVANGÉLICA), Anápolis 75083-515, GO, Brazil; andressamaysapiri@gmail.com (A.M.S.M.); muriloveloso22@gmail.com (M.A.R.B.); silvinhalinda@icloud.com (S.P.R.O.); 3 Health Sciences Graduate Program (PPGCS), Faculty of Medical Sciences (FCMSCSP), Irmandade da Santa Casa de Misericórdia de São Paulo, São Paulo 01221-010, SP, Brazil; tiago.vieirafernandes@hotmail.com (T.V.F.); carloshmendes@unievangelica.edu.br (C.H.M.S.); 4 Department Rehabilitation, Barretos Cancer Hospital, Barretos 14784-400, SP, Brazil; dgmarconi@gmail.com; 5 Professional Master’s Program in Health Innovation, Hospital de Câncer de Barretos, Barretos 14784-400, SP, Brazil; 6 Graduate Program in Dentistry (PPGO), Evangelical University of Goiás (UNIEVANGELICA), Anápolis 75083-515, GO, Brazil; orlandoaguedes@gmail.com (O.A.G.); lucianapmaia@gmail.com (L.P.M.); 7 Orthopaedic Rehabilitation Unit, Research Laboratory for Biomechanics, Rehabilitation and Ergonomics, Ospedale San Giuseppe, 20123 Milan, Italy; p.capodaglio@auxologico.it; 8 Physical and Rehabilitation Medicine Sector, Department of Biomedical, Surgical and Dental Sciences, University of Milan, 20122 Milan, Italy

**Keywords:** physiotherapy, rehabilitation virtual reality, older adults, postural balance

## Abstract

Background: Population aging has been accompanied by an increased incidence of falls, fractures, and functional disability, representing a major global public health challenge. In this context, virtual reality (VR)-based rehabilitation has emerged as an innovative therapeutic strategy providing multimodal sensory and motor stimulation, facilitating motor learning, and enhancing treatment adherence through gamified, interactive environments. Objective: To investigate the effectiveness of VR-based rehabilitation on postural balance training in older adults through a systematic review. Methods: This systematic review was conducted in accordance with PRISMA 2020 and prospectively registered in PROSPERO (CRD420261303119). Comprehensive literature searches were performed in PubMed, Scopus, Web of Science, LILACS, and SciELO to identify studies published between 2015 and 2026. Randomized and non-randomized clinical trials evaluating VR rehabilitation in adults aged 60 years and older, with postural balance as the primary outcome, were eligible. Methodological quality was assessed using the PEDro Scale, and certainty of evidence was evaluated using GRADE. Data were synthesized using a qualitative, descriptive approach. Results: Nine clinical trials of moderate-to-good methodological quality were included, evaluating non-immersive commercial exergames, semi-immersive, and immersive rehabilitation-dedicated systems. Overall, findings indicated that VR was associated with improvements in static and dynamic balance and functional mobility. Between-group analyses showed no statistically significant differences between VR and conventional rehabilitation in several trials, whereas superior effects for VR were reported in others, alongside greater participant engagement and adherence. Conclusions: VR-based rehabilitation is an effective intervention for improving static and dynamic balance and functional mobility in older adults. As a complementary strategy in geriatric physiotherapy, VR has the potential to reduce fall risk, preserve functional independence, and enhance rehabilitation outcomes. Nevertheless, additional high-quality randomized controlled trials with standardized protocols and long-term follow-up are warranted to strengthen the current evidence.

## 1. Introduction

Population aging is a global demographic phenomenon characterized by a structural shift in population age distributions resulting from declining fertility rates and increasing life expectancy, which reached 72.3 years worldwide during the 2015–2020 period [1,2]. According to the United Nations Population Division, individuals aged 65 years and older accounted for 9% of the global population in 2019, and this number is projected to increase to approximately 1.5 billion by 2050, representing 16% of the world’s population [3].

Advancing age is associated with progressive physiological changes, particularly the decline in skeletal muscle mass and muscle strength resulting from the loss and atrophy of muscle fibers. When this process becomes pathological, it is defined as sarcopenia, a geriatric syndrome strongly associated with physical disability, frailty, falls, and other adverse health outcomes [4]. Within this context, falls constitute a major public health concern, as they are closely associated with fractures, functional decline, loss of independence, institutionalization, and increased healthcare expenditures, thereby imposing a substantial socioeconomic burden on healthcare systems worldwide [5,6].

Falls in older adults result from multifactorial causes, including declines in muscle strength, gait, cognitive function, and environmental hazards [6]. Within this context, instability of the body’s center of gravity is a major and modifiable risk factor, as postural balance depends on the coordinated integration of the vestibular, somatosensory, and visual systems through central nervous system mechanisms of sensory reweighting [7,8,9,10]. Impairments in these systems compromise postural control and increase the likelihood of falls during daily activities. To identify and quantify these deficits, validated clinical assessment tools, including the Timed Up and Go (TUG) test, the Berg Balance Scale (BBS), and the Mini-Balance Evaluation Systems Test (MiniBESTest), are widely used in both clinical practice and research because of their reliability, validity, and responsiveness in the geriatric population [10,11,12].

Against this background, physiotherapy plays a pivotal role in the prevention, maintenance, and restoration of functional capacity in older adults [13,14]. Conventional rehabilitation programs are primarily based on muscle strengthening, mobility training, and postural control exercises, with their effectiveness being further enhanced when combined with resistance and endurance training within multicomponent intervention protocols [15]. Nevertheless, adherence to conventional rehabilitation programs is often suboptimal because of the repetitive and monotonous nature of treatment sessions, resulting in progressive participant dropout. This limitation has stimulated the development and implementation of innovative technology-assisted rehabilitation strategies aimed at increasing patient engagement and improving therapeutic outcomes [16].

In this context, virtual reality (VR) has emerged as an innovative rehabilitation modality that simulates interactive virtual environments while enabling continuous monitoring and quantitative assessment of motor performance [17,18,19]. Currently, widely used VR platforms include the Nintendo Wii, Xbox Kinect, Cave Automatic Virtual Environment (CAVE) systems, and head-mounted display devices such as Oculus Quest and HTC Vive. Virtual reality is based on the premise that multimodal sensory stimulation enhances synaptic and cortical neuroplasticity, thereby facilitating motor relearning and functional recovery. According to the degree of user interaction, VR systems are classified as non-immersive, semi-immersive, or fully immersive [20,21,22,23].

Unlike conventional rehabilitation, VR-based interventions enable dynamic adjustment of task difficulty, provide real-time biofeedback, and facilitate high-intensity, repetitive, task-oriented practice, all of which are considered fundamental principles of motor learning and neurorehabilitation [24,25]. Emerging evidence suggests that exergaming interventions may improve static and dynamic balance, muscle strength, postural stability, and functional performance in older adults, with some studies reporting outcomes comparable to those achieved with conventional rehabilitation programs [26,27]. Furthermore, the interactive and gamified nature of VR has been proposed to positively influence cognitive and psychological domains by increasing motivation, enjoyment, and adherence to rehabilitation, and preliminary findings indicate a favorable safety profile with a low incidence of adverse events [28,29,30,31].

Although previous systematic reviews have examined the effects of exergaming and VR on general fall prevention, health-related quality of life, and broad functional outcomes in older adults [24,27], none has specifically targeted postural balance as the primary outcome in adults aged 60 years and older. Existing syntheses either included mixed clinical populations, addressed rehabilitation goals beyond postural control, or treated balance as a secondary measure within broader functional assessments. Furthermore, no previous review has differentiated the comparative effects of non-immersive, semi-immersive, and fully immersive VR systems on postural balance. Therefore, the present systematic review was conducted to evaluate the comparative effectiveness of VR-based rehabilitation versus conventional approaches for postural balance training in this population, with explicit stratification by immersion level and systematic appraisal of evidence certainty.

## 2. Materials and Methods

**Figure 1:**
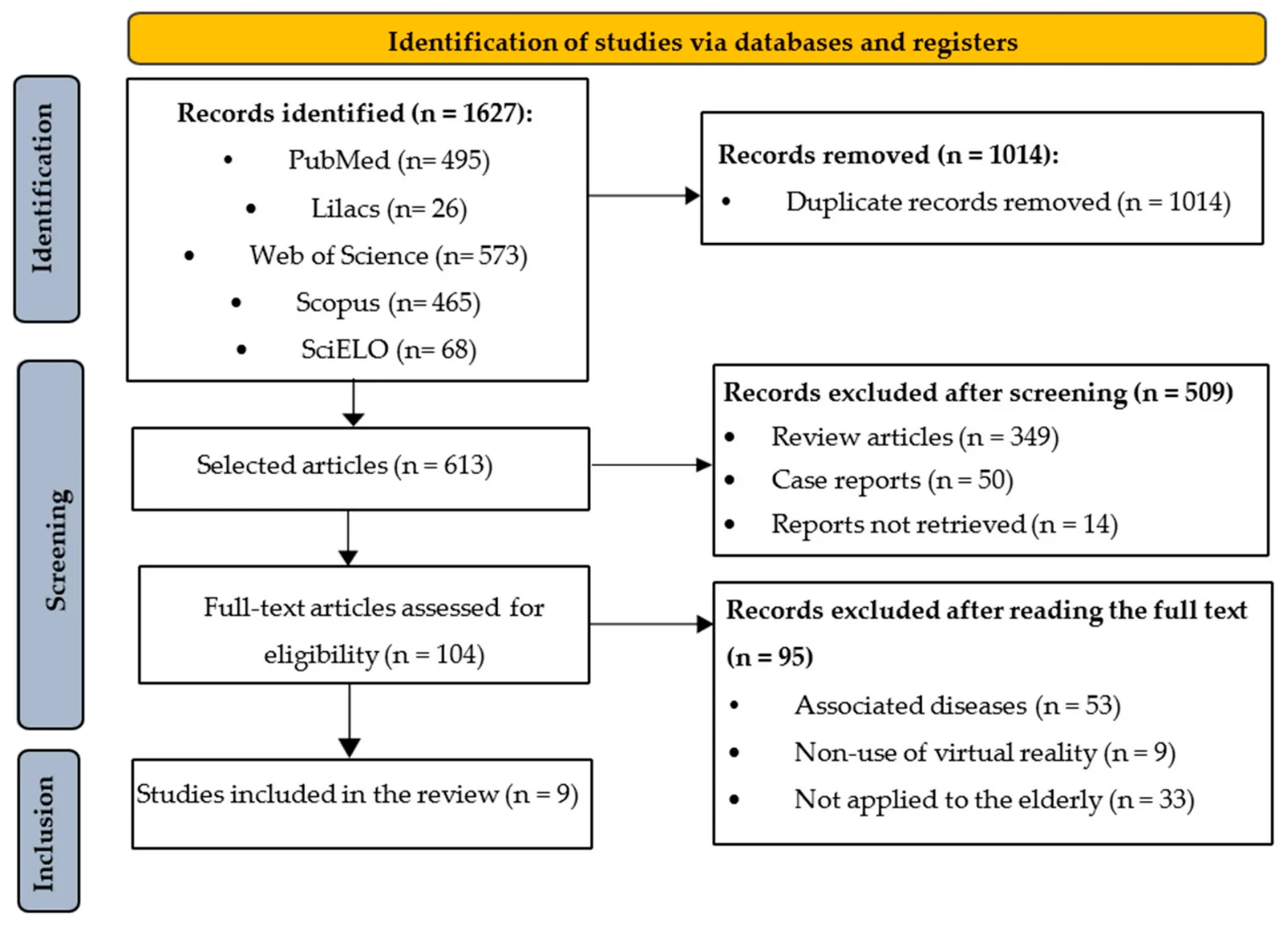
Flow diagram of the systematic review conducted according to the Preferred Reporting Items for Systematic Reviews and Meta-Analyses (PRISMA) guidelines.

This systematic review was conducted in accordance with the Preferred Reporting Items for Systematic Reviews and Meta-Analyses (PRISMA) Statement. The study selection process and review workflow are presented in Figure 1 [32]. In addition, compliance with the PRISMA 2020 reporting guidelines was verified using the corresponding checklist, which is provided as Supplementary Material S1 and S2.

### 2.1. Study Registration and Research Question

The protocol for this systematic review was prospectively registered in the International Prospective Register of Systematic Reviews (PROSPERO) under registration number CRD420261303119 and is publicly available through the PROSPERO database.

The research question was formulated to determine whether VR-based physiotherapy interventions provide greater improvements in postural balance among older adults compared with conventional rehabilitation or other therapeutic approaches. To ensure a structured and transparent review process, the research question and eligibility criteria were defined according to the PICOS framework, as follows:

1. P (Population): Older adults aged 60 years and above.
2. I (Intervention): Physiotherapy interventions based on virtual reality.
3. C (Comparison): Control groups receiving conventional rehabilitation, alternative therapeutic interventions, or no intervention.
4. O (Outcome): Improvement in postural balance.
5. S (Study Design): Clinical trials.

### 2.2. Eligibility Criteria

The eligibility criteria were established according to the PICOS framework. The primary intervention of interest was VR-based rehabilitation delivered through interactive gaming platforms (exergames), which could be implemented either as a standalone intervention or as an adjunct to conventional physiotherapy. On this basis, interactive gaming platforms such as the Nintendo Wii and Xbox Kinect were classified as non-immersive virtual reality (exergaming), because, although the user remains aware of the real environment, their movements are tracked and translated into actions within a virtual environment, and the interaction cycle (action-feedback-adaptation) constitutes the same underlying therapeutic principle as immersive systems.

The included VR systems were classified according to the degree of immersion as follows: (i) non-immersive VR/exergaming: commercial platforms (Nintendo Wii Fit^®^, Xbox Kinect Adventures^®^, MIRA^®^) and interactive projection systems (BTS NIRVANA^®^), in which the user interacts with a virtual scenario displayed on a screen while remaining fully aware of the physical environment; (ii) semi-immersive VR: head-mounted display systems with limited field of view or partial environmental occlusion (Carl Zeiss VR One^®^); and (iii) fully immersive VR: head-mounted displays with wide field of view and full environmental occlusion (Oculus Rift S^®^, Samsung Gear VR^®^).

Randomized and non-randomized clinical trials published between 2015 and 2026 in English, Portuguese, or Spanish were considered eligible. This initial cutoff was defined pragmatically to keep the review of equipment grounded in recent evidence and current clinical-use technologies, as platforms commercialized before this period may differ from current systems in terms of experimentation and tracking specifications. Studies that directly investigated the effectiveness of VR interventions for improving postural balance in adults aged 60 years or older were included.

Studies were excluded if they were duplicate publications, lacked full-text availability, or presented methodological inconsistencies identified during the full-text assessment. In addition, studies that did not meet the predefined eligibility criteria regarding population, intervention, outcomes, or study design were excluded from the qualitative synthesis.

### 2.3. Search Strategy

We conducted the final systematic literature search on 29 May 2026, in the following electronic databases: PubMed, Scopus, Web of Science, the Latin American and Caribbean Health Sciences Literature (LILACS), and the Scientific Electronic Library Online (SciELO). The search strategy was developed using controlled vocabulary based on the Medical Subject Headings (MeSH) database, combined with appropriate Boolean operators (AND and OR) to maximize search sensitivity and specificity. The complete search strategy for each database is presented in Table 1.

Automatic filters were applied to restrict the search to studies published in English, Portuguese, or Spanish between 2015 and 2026. The study selection process was performed independently in three sequential stages: screening of titles, assessment of abstracts, and full-text evaluation of potentially eligible articles. Only studies meeting all predefined eligibility criteria were included in the final qualitative synthesis.

### 2.4. Study Selection

The study selection process was initiated using the Rayyan Systematic Review Accelerator (SRA) to identify potentially duplicate records following the export of bibliographic references [33]. The software automatically generated a separate file containing records suspected of duplication, which were subsequently verified manually. Duplicate citations were identified based on bibliographic information, including author names, article titles, and publication year, and were removed after confirmation of duplication.

Following the removal of duplicate records, study selection was performed in two sequential phases. During the first phase, two independent reviewers (B.O.M.N. and M.C.O.) screened the titles and abstracts of all retrieved records to identify potentially eligible studies according to the predefined eligibility criteria. Articles meeting the inclusion criteria were selected for full-text assessment. In the second phase, the same reviewers independently evaluated the full texts of all potentially eligible studies to confirm their inclusion in the systematic review. Any disagreements regarding study eligibility were resolved through discussion with a third reviewer (L.V.F.O.) until consensus was reached.

### 2.5. Data Extraction

Data extraction was performed independently by two reviewers using a standardized data extraction form, followed by a comparison of the collected information to ensure accuracy and consistency. Any discrepancies were resolved through discussion, and when consensus could not be reached, a third reviewer was consulted to make the final decision. This process was implemented to minimize the risk of extraction errors and reviewer bias.

The following data were extracted from each included study: first author, year of publication, country of origin, study design, sample characteristics (number of participants and age range), characteristics of the intervention (VR platform or exergames used, session frequency, and intervention duration), outcome assessment instruments, primary outcome (postural balance), and the main study findings. Methodological quality was also assessed during this stage to ensure the appropriate application of the evaluation criteria established by the PEDro scale [34].

### 2.6. Methodological Quality Assessment

The methodological quality of the included studies was assessed using the PEDro scale, which consists of 11 items, of which 10 contribute to the final score [34]. The PEDro scale evaluates key methodological domains, including random allocation, allocation concealment, blinding of participants, therapists, and assessors, intention-to-treat analysis, between-group statistical comparisons, and the adequacy of outcome reporting. Total scores range from 0 to 10, with higher scores indicating greater methodological rigor and a lower risk of bias. Studies achieving a PEDro score of ≥5 were considered to be of moderate-to-high methodological quality. This assessment was conducted to inform the interpretation of findings and the GRADE-based certainty of evidence classification.

### 2.7. Data Synthesis

A meta-analysis was considered inappropriate because of the substantial methodological heterogeneity among the included clinical trials. The main sources of heterogeneity included the diversity of VR systems employed (commercial exergaming platforms and customized immersive VR systems), differences in intervention protocols (session frequency, duration, and overall treatment period), and variability in the instruments used to assess postural balance and muscle strength. Consequently, a quantitative synthesis of the findings was not feasible. Therefore, the evidence was synthesized using a qualitative and descriptive (narrative) approach. The results were organized into structured tables and grouped according to the primary outcome (postural balance) and the characteristics of the rehabilitation interventions. This approach enabled a systematic comparison of the included studies and a critical appraisal of the effectiveness of VR-based rehabilitation for improving postural balance in older adults while considering the methodological and clinical diversity across studies.

### 2.8. Certainty of the Evidence for the Outcomes

The certainty of evidence for each outcome was assessed independently by two reviewers using the Grading of Recommendations Assessment, Development and Evaluation (GRADE) approach [35]. Because quantitative synthesis was not performed, imprecision and publication bias were evaluated qualitatively, considering sample sizes, consistency of between-group effects, and breadth of the literature search. Detailed GRADE assessments are provided in Supplementary Material S3.

## 3. Results

### 3.1. Characteristics of the Included Studies

A total of 1627 records related to VR-based postural balance training in older adults were initially identified through the systematic literature search. Following the screening of titles and abstracts and the rigorous application of the predefined inclusion and exclusion criteria, nine clinical trials met the eligibility requirements and were included in the final qualitative synthesis.

The included studies investigated a broad range of VR-based rehabilitation interventions and technological platforms, encompassing commercial exergaming systems as well as customized virtual reality environments designed to improve postural control. Despite variations in intervention protocols and assessment methods, the overall findings consistently demonstrated favorable effects of VR-based rehabilitation on postural balance and functional performance in older adults. Collectively, these studies provide evidence supporting the potential of VR as an effective therapeutic approach for enhancing balance and promoting functional independence in the geriatric population.

### 3.2. Characteristics of the Study Participants

The nine studies included in this systematic review comprised a total of 402 participants, with sample sizes ranging from 18 to 75 individuals per study. The mean age of the participants was 73.6 ± 5.7 years, with reported mean ages ranging from 69.3 to 82.4 years. Five of the nine studies specifically enrolled older adults with a history of falls and/or an increased risk of falling, representing populations at greater risk of functional decline and mobility impairment.

Among the included studies, three investigated institutionalized older adults with a previous history of falls, one evaluated community-dwelling older adults presenting with balance impairments and an elevated risk of falls, and another included participants with chronic dizziness. Three studies recruited healthy or physically active community-dwelling older adults without specific neurological disorders. In addition, one study exclusively enrolled older men to investigate the effects of different exercise modalities on fall-related risk factors.

Overall, the included studies applied rigorous exclusion criteria to enhance participant safety and reduce potential sources of bias. Individuals with cognitive impairment, neurological disorders, severe visual or vestibular deficits, unstable cardiovascular disease, recent fractures or orthopedic surgery, significant joint mobility limitations, dependence on walking aids, inability to ambulate independently, use of medications known to affect postural control, or previous participation in structured balance rehabilitation programs were excluded. These criteria contributed to greater methodological homogeneity and minimized potential confounding factors that could influence postural balance outcomes.

### 3.3. Assessment of Methodological Quality

The methodological quality of the studies included in this systematic review was assessed using the PEDro scale, a widely used instrument for evaluating the internal validity of clinical trials. Given that the nine included studies pertain to the fields of physiotherapy and rehabilitation, the PEDro scale proved adequate for the methodological assessment of the selected studies. Overall, PEDro scores ranged from 5 to 7 points, indicating moderate-to-good methodological quality across the included clinical trials. The methodological domains most consistently fulfilled were random allocation, baseline comparability between groups, and adequate reporting of statistical outcomes, reflecting satisfactory adherence to several key criteria for internal validity.

In contrast, the PEDro items related to the blinding of participants, therapists, and outcome assessors were the least frequently satisfied. This finding is consistent with the inherent methodological challenges associated with physiotherapy and rehabilitation research, where blinding is often impractical because of the nature of the interventions. A detailed summary of the methodological quality assessment and PEDro scores for each included study is presented in Table 2.

### 3.4. Certainty of the Evidence

According to the GRADE framework, the overall certainty of the evidence ranged from low to moderate. The certainty ratings were primarily downgraded due to methodological limitations (lack of blinding and small sample sizes), inconsistency (variability in effect directions across diverse VR platforms and populations), and imprecision (wide confidence intervals). Publication bias could not be reliably evaluated statistically due to the limited number of studies; this domain was qualitatively assessed and did not result in additional downgrading for most outcomes.

### 3.5. Outcome Measures

Across the included studies, postural balance was assessed using a variety of validated clinical instruments, including the BBS, TUG test, Functional Reach Test (FRT), Mini-Balance Evaluation Systems Test (Mini-BESTest), Four Square Step Test (FSST), Limits of Stability (LOS), Single-Leg Stance (SLS) with eyes open and eyes closed, Tandem Stance with Eyes Closed (TS-EC), and the Performance-Oriented Mobility Assessment (POMA). For clarity, the outcomes reported by the included trials were grouped into three categories: (i) balance measures (BBS, Mini-BESTest, POMA, SLS, TS-EC, LOS, FSST, and postural sway); (ii) functional mobility measures (TUG, FRT); and (iii) estimated fall-risk measures (the Physiological Profile Assessment, PPA, and functional tests such as the TUG used as indirect fall-risk indicators). None of the included studies directly measured fall incidence as an outcome; therefore, statements regarding fall prevention in this review refer exclusively to improvements in balance and mobility measures that are associated with fall risk, and not to a demonstrated reduction in actual falls.

Most studies reported significant within-group improvements in balance and functional mobility following VR-based rehabilitation. However, significant within-group changes do not, in isolation, establish therapeutic superiority; accordingly, the following synthesis prioritizes between-group comparisons where available. Between-group analyses indicated that VR-based interventions produced statistically superior outcomes compared with conventional rehabilitation in several trials [7,8,37,39], whereas other trials reported no statistically significant differences between the interventions [30,40], a finding that should not be interpreted as evidence of equivalence, given the small sample sizes and limited statistical power of those trials.

The most consistently reported favorable outcomes included improvements in static and dynamic postural balance, as demonstrated by increased scores on the BBS, Mini-BESTest, POMA, and single-leg and tandem stance tests [7,30,37,38]. In addition, several studies reported reductions in TUG completion time, indicating improved functional mobility [7,37,39].

When the findings were examined according to the degree of immersion (Table 3), non-immersive platforms (Nintendo Wii Fit^®^, Xbox Kinect^®^, MIRA^®^, BTS NIRVANA^®^) demonstrated positive effects on postural balance and functional mobility [30,36,37,39,41], with no statistically significant between-group differences in favor of VR or conventional training in one trial [30]. The only semi-immersive system evaluated (Carl Zeiss VR One^®^) produced within-group improvements, particularly when combined with dual-task training [7]. Fully immersive systems (Oculus Rift S^®^, Samsung Gear VR^®^) also improved balance and functional mobility [7,8]; however, in one trial these benefits were not statistically superior to those achieved with conventional physiotherapy [40]. Taken together, these findings indicate that immersive VR represents a viable therapeutic alternative but does not consistently outperform established rehabilitation strategies.

In the studies that added VR to another active component, dual-task training combined with semi-immersive VR [7], Kinect-based exergaming combined with reaction-based games [38], and vestibular rehabilitation combined with 360° real-life virtual environments [8]. The combined or enriched conditions reported improvements in functional performance, postural stability, and mobility. However, because these trials did not include a group receiving VR alone, the additional gains cannot be attributed exclusively to VR; they may reflect the accompanying component, the greater total exercise dose, or a genuine synergistic effect. These findings are consistent with the hypothesis that increasing cognitive and environmental demands during rehabilitation may enhance motor learning and promote the transfer of acquired skills to activities of daily living, but this question should be directly tested in future trials with appropriate comparison groups.

Follow-up data were available in only two of the included studies (22%) [8,30], according Table 4. This scarcity of long-term evidence precludes any conclusion regarding the persistence or durability of treatment effects beyond the post-intervention period. In the study by Bacha et al. [30], the improvements in Mini-BESTest scores observed immediately after treatment were maintained at the four-week follow-up in both the VR and the conventional physiotherapy groups, indicating short-term preservation of dynamic balance gains regardless of the rehabilitation modality. Kanyılmaz et al. [8] extended the follow-up to six months and reported that the between-group difference in BBS scores in favor of the VR-assisted vestibular rehabilitation group remained statistically significant at that time point (*p* = 0.042). However, because this longer-term evidence derives from a single small trial, it should be regarded as preliminary and does not allow conclusions on the durable long-term benefits of VR-based rehabilitation.

## 4. Discussion

The studies included in this systematic review demonstrate that VR has emerged as a multifaceted intervention in geriatric physiotherapy rehabilitation, eliciting consistent within-group improvements in postural balance and functional mobility. Between-group effects were not significantly different from, or were superior to, those achieved with conventional rehabilitation approaches. The therapeutic advantages of VR appear to result from the integration of physical exercise with cognitive challenges, creating an enriched rehabilitation environment that promotes neuroplasticity while enhancing patient engagement and adherence through increased motivation and interactive feedback [7,30,36,37,38,39,41,42].

Conventional physiotherapy is based on the direct delivery of therapist-guided interventions, including neuromuscular facilitation techniques, therapeutic stretching, resistance training, functional exercises, and individualized biomechanical correction. Its therapeutic effectiveness relies on the provision of accurate proprioceptive input, which facilitates sensorimotor reorganization and the refinement of movement patterns. This approach emphasizes guided postural control, supervised motor relearning, and individualized progression of therapeutic exercises, and has consistently demonstrated effectiveness in reducing fall risk, restoring functional capacity, and improving muscle strength and mobility in older adults [42,43,44].

In contrast, VR-based physiotherapy employs interactive digital technologies to facilitate motor rehabilitation through the integration of visual, auditory, vestibular, and motor stimuli within immersive or non-immersive environments. This multisensory stimulation enhances cognitive engagement and increases cortical motor drive during task execution. Unlike conventional rehabilitation, motor skill acquisition is primarily based on error-driven learning supported by real-time feedback and the progressive adjustment of task difficulty, thereby promoting neuromotor adaptation and experience-dependent neuroplasticity. Furthermore, the gamified and interactive nature of VR interventions increases motivation, training intensity, and adherence to rehabilitation while simultaneously challenging higher-order cognitive functions, including sustained attention, reaction time, executive function, and visuospatial coordination [42,45,46].

When compared with conventional rehabilitation, the studies by Yeşilyaprak et al. [37], Bacha et al. [30], and Żak et al. [7] demonstrated that both approaches are effective in improving balance and functional performance in older adults. However, VR-based rehabilitation appears to offer additional advantages, particularly with respect to patient engagement, motivation, and adherence to treatment. These findings are supported by Stanmore et al. [41], who reported higher adherence rates among participants enrolled in rehabilitation programs incorporating exergames. Similarly, Song et al. [47] emphasized that the integration of visual, auditory, and cognitive stimuli increases participant engagement and promotes active involvement throughout the rehabilitation process. Collectively, the available evidence suggests that VR should not be viewed as a replacement for conventional physiotherapy but rather as a complementary therapeutic modality capable of enhancing the effectiveness of traditional rehabilitation by incorporating motivational and interactive elements that encourage greater participation and sustained training intensity [18].

From a clinical perspective, these findings suggest that VR-based rehabilitation can be integrated into geriatric physiotherapy protocols as a complementary strategy to enhance patient engagement without compromising therapeutic efficacy [18,24,43]. The ability to individualize task difficulty and provide real-time feedback makes VR particularly suitable for older adults with varying degrees of functional impairment [7,30]. However, technology selection should be guided by the patient’s sensory profile, cognitive status, and accessibility, given the differential effects observed across immersive and non-immersive systems [8,41].

The observed improvements in balance and functional mobility may be partially attributed to neuroplastic adaptations induced by repetitive, multisensory motor training. However, this interpretation remains speculative, as none of the included studies directly assessed neurophysiological mechanisms [44,48,49,50,51,52].

The cognitive dimension of VR-based rehabilitation has also been consistently highlighted in the broader literature. Studies by Lopes et al. [43] and Song et al. [47] emphasized that interactive virtual environments strongly stimulate executive functions, including decision-making, working memory, and sustained attention, throughout task execution. Although cognitive outcomes were not directly assessed in most of the studies included in this review, these mechanisms are indirectly supported by reports of greater participant engagement and superior functional performance, particularly in the studies conducted by Stanmore et al. [41] and Bacha et al. [30]. The simultaneous integration of physical and cognitive challenges within a single therapeutic environment may enhance motor learning, promote functional independence, and improve performance in ADLs, thereby reinforcing the multidimensional benefits of VR-based rehabilitation for older adults.

Although the included studies did not systematically evaluate motivation, engagement, or adherence as measured outcomes, feasibility assessments reported by Stanmore et al. [41] suggest that the interactive nature of exergames may positively influence treatment adherence. These observations are consistent with broader literature indicating that gamified rehabilitation environments can enhance patient satisfaction and perceived functional improvement [47]. Nevertheless, given the absence of validated instruments assessing these constructs across the included trials, conclusions regarding the consistent enhancement of motivation and adherence cannot be drawn from the current evidence base.

An analysis of the intervention protocols revealed that most studies implemented VR-based rehabilitation programs lasting between 3 and 8 weeks, with treatment sessions ranging from 30 to 60 min. Despite these relatively short intervention periods, significant improvements in postural balance, muscle strength, and functional performance were consistently reported, as demonstrated by Bacha et al. [30], Fu et al. [36], and Sadeghi et al. [39]. These findings are supported by the systematic review conducted by Cacciata et al. [24], which suggested that relatively brief interventions can induce meaningful neuromotor adaptations when combined with intensive multisensory stimulation. Collectively, these results indicate that VR-based rehabilitation can produce clinically relevant improvements within a relatively short timeframe, supporting its feasibility and applicability across a wide range of geriatric rehabilitation settings.

Regarding safety, all studies included in this systematic review reported a low incidence of adverse events, with only mild and transient discomfort being observed during VR-based interventions. These findings are consistent with the systematic reviews by Chen et al. [48] and Cacciata et al. [24], both of which concluded that virtual reality is a safe rehabilitation modality for older adults. The ability to adjust task difficulty according to individual capabilities and to monitor performance in real time enables therapists to maintain greater control over the rehabilitation environment while minimizing potential risks. Consequently, VR-based rehabilitation appears to be a safe therapeutic option, even for older adults with significant functional impairments [30].

Several methodological limitations were identified across the included studies, most notably the relatively short follow-up periods and the small sample sizes. Similar limitations have been reported in previous systematic reviews, including those by Cacciata et al. [24] and Chen-PJ et al. [48], suggesting that the current evidence base for VR-based rehabilitation is still evolving. These observations underscore the need for large-scale, multicenter randomized controlled trials with standardized intervention protocols and extended follow-up periods to establish the long-term effectiveness of VR interventions. Nevertheless, the consistent demonstration of favorable outcomes across studies with diverse methodological designs strengthens the overall evidence supporting the use of VR as an effective rehabilitation strategy for improving balance and functional mobility in older adults.

When the different VR systems were compared according to the classification adopted in this review, non-immersive exergaming platforms (Nintendo Wii Fit^®^ [36], BTS NIRVANA^®^ [37], Kinect^®^ [30,38,39], and MIRA^®^ [41]), semi-immersive systems (Carl Zeiss VR One^®^ [7]), and fully immersive head-mounted displays (Oculus Rift S^®^ [7,40] and Samsung Gear VR^®^ [8]) were all associated with significant improvements in balance-related outcomes, although the magnitude of the effects varied according to the clinical population and intervention characteristics.

For example, Kanyılmaz et al. [8] reported particularly favorable outcomes among older adults with vestibular disorders, whereas Fu et al. [36] and Sápi et al. [38] demonstrated greater effectiveness in improving fall-related risk measures. Likewise, the systematic review by Intziegianni et al. [46] suggested that immersive VR platforms may produce broader benefits in sensory integration than non-immersive systems. These findings indicate that the selection of VR technology should be individualized according to the clinical characteristics, functional impairments, and rehabilitation goals of each older adult.

The clinical effectiveness of VR-based rehabilitation is closely associated with the integration of inertial sensors, three-dimensional motion capture systems, and wearable technologies, which enable continuous kinematic monitoring and real-time quantification of biomechanical and functional performance variables. Adaptive algorithms applied to these data facilitate individualized progression of rehabilitation protocols, thereby improving treatment precision, methodological rigor, and intervention reproducibility. Furthermore, digital rehabilitation technologies have the potential to expand access to specialized rehabilitation services, particularly for individuals living in remote or underserved regions and for vulnerable populations, including older adults and patients with chronic neurological disorders. By reducing geographical and logistical barriers, these technologies also support the implementation of telerehabilitation programs and long-term remote monitoring, thereby extending the continuity and accessibility of evidence-based rehabilitation services [49,50,53].

### Strengths and Limitations

This review has several strengths. The protocol was prospectively registered in PROSPERO (CRD420261303119), and the review was conducted in accordance with the PRISMA 2020 guidelines. A comprehensive literature search was performed across major electronic databases, and study selection and data extraction were performed independently by two reviewers to minimize bias. Methodological quality was assessed using the PEDro scale, and the certainty of the evidence was evaluated using the GRADE approach, enhancing the rigor and transparency of the synthesis.

This review also has limitations. The literature search was restricted to studies published in English, Portuguese, or Spanish, which may have influenced the representativeness of the evidence identified. The included studies were characterized by small sample sizes, short follow-up periods, lack of blinding, and substantial heterogeneity in VR systems, intervention protocols, and outcome instruments, which precluded quantitative synthesis and limited direct comparability across trials. Furthermore, the study populations encompassed diverse groups of older adults, including physically active community-dwelling individuals, institutionalized older adults with a history of falls, patients with vestibular disorders, and individuals presenting with functional decline, thereby limiting the generalizability of findings to specific subgroups.

Consequently, future research should prioritize large-scale, multicenter randomized controlled trials with standardized protocols to enable meta-analytic pooling [24,31]. Studies should incorporate direct fall incidence as a primary outcome, alongside balance measures, to establish a clearer causal pathway between VR-based training and fall prevention [5,6]. Additionally, investigations comparing the cost-effectiveness of immersive versus non-immersive systems, as well as the optimal dosage and long-term sustainability of treatment effects, are warranted to inform evidence-based clinical guidelines [46,48].

## 5. Conclusions

The findings of this systematic review suggest that VR-based rehabilitation may be a promising therapeutic strategy for postural balance training in specific subgroups of older adults. The available evidence indicates within-group improvements in static and dynamic balance and functional mobility following VR-based interventions across diverse clinical populations and VR platforms. Between-group analyses further suggest that VR produces effects that did not differ significantly from, or were superior to, conventional rehabilitation in several trials, although these findings should be interpreted with caution given the heterogeneity of participant characteristics, VR systems, and intervention protocols.

The various VR technologies evaluated, including non-immersive, semi-immersive, and immersive systems, proved effective across different clinical settings, although their applicability may vary according to the functional status and clinical characteristics of older adults. Furthermore, the interactive and gamified nature of VR may enhance motivation, engagement, and adherence to rehabilitation, although these outcomes were not systematically assessed across the included studies. The current evidence remains limited by methodological heterogeneity and the relatively small number of high-quality clinical trials. Future well-designed randomized controlled trials with standardized intervention protocols, larger sample sizes, and long-term follow-up are warranted to strengthen the evidence base and establish the optimal clinical application of VR-based rehabilitation in the geriatric population.

## Figures and Tables

**Table 1:** Electronic databases and search strategies.

Databases	Search Strategies
Pubmed	((“Virtual Reality”[Mesh] OR “virtual reality” OR “virtual rehabilitation” OR exergaming OR “video game therapy” OR “virtual physiotherapy”)) AND ((“Aged”[Mesh] OR elderly OR “older adults” OR geriatric)) AND ((“Postural Balance”[Mesh] OR balance OR “postural control”)) AND ((“Rehabilitation”[Mesh] OR “Physical Therapy Modalities”[Mesh] OR physiotherapy OR “physical therapy”))
Scopus	TITLE-ABS-KEY ((“virtual reality” OR “virtual rehabilitation” OR exergaming OR “virtual physiotherapy”) AND (elderly OR aged OR “older adults”) AND (“postural balance” OR balance) AND (rehabilitation OR physiotherapy OR “physical therapy”))
Web of Science	((“virtual reality” OR “virtual rehabilitation” OR exergaming OR “virtual physiotherapy”) AND (“older adults” OR elderly OR aged) AND (“postural balance” OR balance) AND (rehabilitation OR physiotherapy OR “physical therapy”))
Lilacs	((“realidade virtual” OR “reabilitação virtual” OR “reabilitação virtual” OR “virtual reality” OR exergame OR exergaming)) AND ((“idosos” OR idoso OR elderly OR aged OR “adultos mayores”)) AND ((“equilíbrio postural” OR balance OR “postural balance”)) AND ((“reabilitação” OR fisioterapia OR rehabilitation OR physiotherapy))
Scielo	((“virtual reality” OR “virtual rehabilitation” OR exergaming OR “video game therapy” OR “virtual physiotherapy”) AND (elderly OR aged OR “older adults” OR geriatric) AND (“postural balance” OR balance OR “postural control”) AND (rehabilitation OR physiotherapy OR “physical therapy”))

**Table 2:** Methodological quality assessment of the included studies according to the Physiotherapy Evidence Database (PEDro) scale.

Study (Author/Year)	1	2	3	4	5	6	7	8	9	10	11	Total
Fu et al., 2015 [36]	Y	Y	N	N	N	N	N	Y	N	Y	Y	5/10
Yeşilyaprak et al., 2016 [37]	Y	Y	N	Y	N	N	N	Y	N	Y	Y	5/10
Bacha et al., 2018 [30]	Y	Y	N	Y	N	N	N	Y	N	Y	Y	5/10
Sápi et al., 2019 [38]	Y	Y	N	Y	N	N	N	Y	N	Y	Y	5/10
Sadeghi et al., 2021 [39]	Y	Y	N	Y	N	N	Y	Y	N	Y	Y	6/10
Rebêlo et al., 2021 [40]	Y	Y	Y	Y	N	N	Y	Y	N	Y	Y	7/10
Zak et al., 2022 [7]	Y	Y	N	Y	N	N	N	Y	N	Y	Y	5/10
Kanyılmaz et al., 2022 [8]	Y	Y	N	Y	N	N	Y	Y	N	Y	Y	6/10
Stanmore et al., 2024 [41]	Y	Y	N	Y	N	N	N	Y	N	Y	Y	5/10

Abbreviations: Y, Yes; N, No.

**Table 3:** Methodological Characteristics and Main Findings of the Selected Clinical Trials.

Author and Year/ Sample	Objective	Intervention	Outcome Measures (Pre- vs. Post-Intervention)	Conclusion
Fu et al. (2015) [36], China (Hong Kong) Sample: 60 community-dwelling older adults aged ≥65 years (mean age: 82.4 years) with a history of falls. Wii Fit Training Group (G1): n = 30 Conventional Balance Training Group (G2): n = 30	To investigate the effectiveness of Nintendo Wii Fit exergaming in reducing fall risk and fall incidence among older adults with a history of falls.	Duration: 6 weeks, three 1 h sessions per week. G1: Nintendo Wii Fit Balance Board (non-immersive/exergaming) using the games Soccer Heading, Table Tilt, and Balance Bubble. G2: Conventional balance training including lower-limb muscle strengthening, weight-shifting and anticipatory postural control exercises, tandem walking, side-stepping, and squats.	Reaction time (ms) (*p* < 0.001): G1: 344.3 ± 77.3 → 315.5 ± 74.2 G2: 346.6 ± 89.0 → 338.9 ± 87.6 Postural sway (mm^2^) (*p* = 0.013): G1: 1364.0 ± 372.5 → 1042.0 ± 317.2 G2: 1213.5 ± 390.7 → 1330.8 ± 510.4 PPA z-score (*p* = 0.004): G1: 3.7 ± 1.0 → 2.4 ± 1.0 G2: 3.7 ± 1.2 → 3.3 ± 1.2	Wii Fit^®^ training significantly improved postural balance, as demonstrated by lower PPA scores, faster reaction time, and reduced postural sway, indicating greater improvements in fall-related risk measures compared with conventional balance training. Between-group analyses favored Wii Fit for fall-related risk measures.
Yeşilyaprak et al. (2016) [37], Türkiye Sample: 18 older adults with a history of falls (65–82 years). VR Group (G1): n = 7 (mean age: 70.1 years) Conventional Group (G2): n = 11 (mean age: 73.1 years)	To investigate the effects of VR-based balance exercises on balance performance and fall risk compared with conventional balance exercises in older adults residing in nursing homes.	Duration: 6 weeks (three 50 min sessions per week). G1: BTS NIRVANA interactive VR system (non-immersive/exergaming). Training consisted of static and dynamic balance tasks projected onto walls or the floor, requiring hand–eye coordination and gaze stabilization while participants interacted with virtual visual targets (e.g., collecting hearts and following butterflies). G2: Functional balance exercises including marching in place, sit-to-stand training, heel raises, tandem walking, and leg-raising exercises, with progressive reduction in the base of support.	BBS (*p* < 0.01): G1: 51.0 ± 2.7 → 54.4 ± 3.4 G2: 49.6 ± 3.1 → 51.8 ± 3.9 TUG (s) (*p* < 0.01): G1: 12.7 ± 6.5 → 9.6 ± 4.0 G2: 11.4 ± 4.1 → 10.0 ± 3.7 OLS-EC-L (s) (*p* < 0.01): G1: 4.7 ± 3.5 → 9.3 ± 5.0 G2: 2.6 ± 1.8 → 3.9 ± 3.5 TS-EC (s) (*p* = 0.04) G1: 5.4 ± 5.0 → 15.7 ± 11.5 G2: 9.2 ± 6.8 → 10.3 ± 5.5	VR-based balance training significantly improved postural balance, as evidenced by higher BBS scores, better functional mobility (TUG), and longer one-leg and tandem stance times with eyes closed. Both VR and conventional balance training effectively improved fall-related risk measures within-group; the VR group demonstrated greater within-group improvements in postural stability.
Bacha et al. (2018) [30], Brazil Sample: 46 older adults (mean age: 69.3 years). VR Group (G1): n = 23 Conventional Physical Therapy Group (G2): n = 23	To compare the effectiveness of Kinect Adventures games versus conventional physical therapy in improving postural control, gait, cardiorespiratory fitness, and cognition in older adults.	Duration: 7 weeks (two 1 h sessions). G1: Kinect Adventures exergames (non-immersive), including Space Pop, 20,000 Leaks, Reflex Ridge, and River Rush. G2: A conventional exercise program consisting of warm-up exercises, static and dynamic balance training, endurance exercises, muscle strengthening, motor coordination exercises, and stretching.	Mini-BESTest (points) (Pre- vs. Post-Intervention) G1: 26.52 ± 3.24 → 29.65 ± 2.87 (*p* < 0.005) G2: 27.60 ± 2.64 → 29.82 ± 2.08 (*p* < 0.005) Between-group mean: 27.06 ± 2.97 → 29.73 ± 2.48 (*p* < 0.001)	No significant differences were observed between the VR and conventional physical therapy groups. Both interventions produced significant improvements in postural control, gait performance, cardiorespiratory fitness, and cognitive function in older adults.
Sápi et al. (2019) [38], Hungary Sample: 75 older adults (mean age: 69.57 years). Kinect Training Group (G1): n = 30 Conventional Training Group (G2): n = 23 Control Group (G3): n = 22	To evaluate whether Kinect-based training is more effective than conventional balance training in improving functional balance and posturographic measures of postural stability assessed through visual feedback.	Duration: 6 weeks (three 30 min sessions). G1: Participants performed Kinect-based exergames (non-immersive), including bowling, skiing, and soccer during the first half of each session, followed by reaction-based games (20,000 Leaks and Space Pop) during the second half. G2: Participants performed a conventional exercise program consisting of warm-up exercises, weight-shifting activities, squats, jumping exercises, and a cool-down period with stretching. G3: Participants maintained their usual daily activities without supervised exercise.	FSST (s) (Pre- vs. Post-Intervention) (*p* < 0.05) Between-group mean difference: 4.37 FRT (cm) (Pre- vs. Post-Intervention) (*p* < 0.05) Between-group mean difference: 3.65 TUG (s) (Time × Group interaction) Mean difference: 3.46 (*p* < 0.05) TUG-DT (s) (Time × Group interaction) Mean difference: 3.48 (*p* < 0.05) LOS-RT (s) (Pre- vs. Post-Intervention) (*p* < 0.01) G1: 1.04 ± 0.63 → 0.89 ± 0.54 G2: 0.94 ± 0.62 → 1.00 ± 0.50 G3: 0.91 ± 0.51 → 1.04 ± 0.62 LOS-V (m/s) (Pre- vs. Post-Intervention) (*p* = 0.007) G1: 3.09 ± 1.38 → 3.84 ± 1.84 G2: 3.55 ± 1.61 → 3.77 ± 4.38 G3: 3.05 ± 1.59 → 2.82 ± 1.15	Kinect^®^-based training significantly improved postural balance, as demonstrated by superior performance on the TUG, FSST, FRT, and LOS tests. These findings were characterized by reduced task completion times and enhanced postural control and stability. The virtual rehabilitation group achieved greater improvements than the conventional training group, supporting the effectiveness of Kinect^®^-based training for enhancing balance and improving performance on fall-related functional measures
Sadeghi et al. (2021) [39], Iran/Malaysia/Canada Sample: 58 older men (mean age: 71.8 years). Traditional Balance Training (BT) (G1): n = 14 VR (G2): n = 15 Combined Training (MIX) (G3): n = 14 Control (G4): n = 15	To determine whether traditional BT, VR, or MIX would produce greater improvements in balance and functional mobility, used as proxies for fall risk, compared with a CG in older men.	Duration: 8 weeks (three 40 min sessions per week). G1: Static and dynamic exercises (e.g., single-leg stance, tandem walking). G2: (1) The Light Race (Stomp It) mini-game from the Your Shape fitness package, (2) the Target Kick mini-game, and (3) the Goalkeeper mini-games from the Sport Xbox Kinect game (non-immersive/exergaming) package. G3: 15 min of BT and 15 min of VR. G4: Maintenance of usual daily activities.	Single-Leg Stance on a Firm Surface (s) (*p* < 0.05) Dominant/Non-dominant leg: G1: −7.2 (−8.7, −5.6)/−6.1 (−7.7, −4.4) G2: −12.7 (−14.8, −10.4)/−11.8 (−13.7, −9.9) G3: −15.0 (−17.0, −13.1)/−15.5 (−17.2, −13.8) G4: 0.7 (−0.03, 1.5)/0.4 (−0.2, 0.9) Single-Leg Stance on a Foam Surface (s) (*p* < 0.05) Dominant/Non-dominant leg: G1: −9.1 (−12.1, −6.1)/−11.3 (−15.5, −7.0) G2: −9.6 (−13.6, −5.5)/−12.2 (−15.6, −8.8) G3: −9.4 (−14.6, −4.3)/−9.9 (−12.9, −6.9) G4: −0.3 (−0.6, 0.04)/0.1 (−1.3, 1.4) Tandem Stance (s) (*p* < 0.05) G1: −7.8 (−9.8, −5.3) G2: −10.2 (−13.1, −7.3) G3: −10.4 (−12.5, −8.4) G4: −0.3 (−0.9, 0.1) TUG (s) (*p* < 0.05) G1: 1.5 (0.8, 2.2) G2: 2.5 (2.0, 3.1) G3: 5.2 (4.7, 5.7) G4: −0.3 (−0.5, 0.1)	The MIX group demonstrated the greatest improvements in balance and functional mobility compared with the BT, VR, and CON groups. The VR group showed greater improvements in balance and functional mobility than the BT and CON groups, whereas the BT group achieved superior outcomes compared with the CON group.
Rebêlo et al. (2021) [40], Brazil Sample: 37 older adults aged 60–79 years with balance impairments and an increased risk of falls. Immersive VR Group (G1): n = 20 Control Group (G2): n = 17	To investigate the effects of immersive VR on the rehabilitation of older adults with balance impairments and an increased risk of falls.	Duration: 8 weeks (two 50 min sessions). G1: Training was performed using the Oculus Rift HMD (fully immersive) with the exergames (BoxVR, Baskhead, InCell, and Thrills and Chills Roller Coasters), focusing on squatting, punching, head movements, and postural. G2: Multimodal exercise circuit (walking, balance training, obstacle negotiation, and trunk stability).	TUG (s) (Mean Change [Post–Pre]) G1: −1.71 G2: −1.22 FRT (cm) (Mean Change [Post–Pre]) G1: 4.30 G2: 8.64	Immersive VR was effective in improving balance-related outcomes within-group; however, no significant between-group superiority over conventional physical therapy was observed.
Zak et al. (2022) [7], Poland/France Sample: 60 older adults (mean age: 77.6 years). Conventional Exercise Program Group (G1): n = 15 Semi-Immersive VR Group (G2): n = 15 Combined Dual-Task and Carl Zeiss VR Group (G3): n = 15 Fully Immersive Oculus Rift S Group (G4): n = 15	To evaluate the effectiveness of VR–assisted physical therapy programs in older adults with functional impairment.	Duration: 3 weeks (three 30 min sessions per week). G1: Balance, flexibility, and resistance training using elastic bands. Balance exercises focused on standing, walking, stair ascent and descent, and sit-to-stand training. G2: Training using the Carl Zeiss VR One HMD (semi-immersive), including navigation through a virtual maze, forward, backward, diagonal, and square walking, squatting, and trunk rotation exercises. G3: A combined intervention consisting of 15 min of dual-task exercises followed by 15 min of VR training using the Carl Zeiss VR One system (semi-immersive). G4: Training using the Oculus Rift S system (fully immersive) within a four-room virtual rehabilitation environment: Room 1—cognitive exercises; Room 2—aerobic exercises; Room 3—static and dynamic balance training; and Room 4—dual-task training activities.	Values are reported as means. TUG (s) (Pre- vs. Post-Intervention) (*p* = 0.001) G1: 13.29 → 11.89 G2: 13.51 → 12.65 G3: 14.75 → 14.19 G4: 15.61 → 14.42 POMA-G (pts) (Pre- vs. Post-Intervention) (*p* = 0.04) G1: 9.60 → 9.73 G2: 7.73 → 8.26 G3: 7.80 → 8.93 G4: 8.26 → 9.06 SLS-EO (s) (Pre- vs. Post-Intervention) (*p* < 0.001) G1: 5.35 → 6.70 G2: 5.61 → 5.75 G3: 20.20 → 24.98 G4: 14.39 → 16.44 SLS-EC (s) (Pre- vs. Post-Intervention) (*p* < 0.001) G1: 1.45 → 1.55 G2: 1.18 → 1.46 G3: 4.05 → 5.06 G4: 8.45 → 9.26	The combined dual-task and VR group demonstrated the greatest improvement in the SLS-EO test. The VR and Oculus groups also showed marked improvements in the SLS-EC test. The Oculus group achieved significantly greater improvements in TUG performance than the VR and conventional exercise groups. The virtual reality–based intervention groups (VR, combined dual-task VR, and Oculus) demonstrated greater improvements in the total POMA score than the conventional exercise group.
Kanyılmaz et al. (2022) [8], Turkey Sample: 26 older adults aged ≥65 years (mean age: 70 years) with chronic dizziness. VR–Assisted Vestibular Rehabilitation Group (G1): n = 13 Conventional Vestibular Rehabilitation Group (G2): n = 13	To investigate the effects of VR–assisted vestibular rehabilitation exercises using real-life environments on dizziness, static and dynamic balance, functional mobility, fear of falling, anxiety, and depression in older adults with dizziness.	Duration: 3 weeks (five 30 min sessions). G1: Vestibular rehabilitation exercises targeting eye movements, gaze stabilization, and postural stability, supplemented with Samsung Gear VR (fully immersive) head-mounted displays and Samsung Galaxy S7 smartphones to present 360° three-dimensional (3D) videos recorded in real-life environments, including: a large public square with heavy pedestrian and vehicular traffic, incorporating authentic environmental sounds; and the aisles of a large supermarket containing shelves stocked with products of varying shapes and colors. G2: Participants performed the same vestibular rehabilitation exercises as the G1 group, without VR.	BBS (pts) (Median [Interquartile Range, IQR]; Pre- vs. Post-Intervention) G1: 50.00 (6.00) → 54.00 (3.00) G2: 51.00 (6.00) → 52.00 (5.00) Between-group comparison: Pre-intervention: *p* = 0.737 Post-intervention: *p* = 0.108 TUG (s) (Median [IQR]; Pre- vs. Post-Intervention) G1: 14.00 (2.50) → 11.00 (1.50) G2: 15.00 (5.00) → 13.00 (4.50) Between-group comparison: Pre-intervention: *p* = 0.286 Post-intervention: *p* = 0.004	The integration of virtual reality environments using 360° 3D videos of real-life settings was superior to conventional vestibular rehabilitation alone. Although both interventions produced beneficial effects, the VR–assisted group demonstrated significantly greater improvements in functional mobility, as assessed by the TUG test, and in emotional status immediately after the intervention.
Stanmore et al. (2024) [41], United Kingdom Sample: 22 older adults (mean age: 74.3 years). Intervention Group (G1): n = 10 CG (G2): n = 12	To evaluate the feasibility, acceptability, and usability of an exergaming intervention in assisted living facilities.	Duration: 6 weeks (three 30 min sessions). G1: MIRA exergaming (non-immersive) program consisting of strength and balance exercises targeting the knee flexors, knee extensors, hip abductors, and ankle dorsiflexor and plantar flexor muscles. G2: Usual care, including standard advice on balance and muscle-strengthening exercises.	BBS (pts) (Pre- vs. Post-Intervention) G1: 39.4 ± 11.3 → 43.0 ± 11.9 G2: 38.7 ± 12.2 → 39.8 ± 10.8 Mean Difference (95% CI): −4.3 (−10.7 to 2.1)	Although the study was underpowered to detect statistically significant differences because of its small sample size, the trends consistently favored the exergaming group across all outcome measures.

Abbreviations: BBS, Berg Balance Scale; BT, Traditional Balance Training; CG, Control Group; CON, Control; FRT, Functional Reach Test; FSST, Four Square Step Test; G1, Group 1; G2, Group 2; G3, Group 3; G4, Group 4; HMD, head-mounted display; IQR, interquartile range; LOS-RT, Limits of Stability—Reaction Time; LOS-V, Limits of Stability—Movement Velocity; Mini-BESTest, Mini-Balance Evaluation Systems Test; MIX, Combined Training; OLS-EC-L, One-Leg Stance, Eyes Closed-Left; POMA-G, Performance-Oriented Mobility Assessment—Gait Subscale; PPA, Physiological Profile Assessment; pts, points; SLS-EC, Single-Leg Stance with Eyes Closed; SLS-EO, Single-Leg Stance with Eyes Open; TS-EC, Tandem Stance, Eyes Closed; TUG, Timed Up and Go; TUG-DT, Timed Up and Go with Dual Task; VR, Virtual reality. Static balance measures: SLS, TS-EC, OLS-EC. Dynamic balance measures: BBS, TUG, FRT, FSST, LOS, Mini-BESTest, POMA. Between-group comparisons (mean differences, 95% confidence intervals, and *p*-values) are reported separately where available.

**Table 4:** Clinical trials reporting follow-up outcomes.

Author (Year)/Sample	Follow-up	Main Results
Bacha et al. (2018) [30] Sample: 46 older adults (mean age: 69.3 years) VR Group (G1): *n* = 23 Conventional Physiotherapy Group (G2): *n* = 23	4-week follow-up	Mini-BESTest score (mean ± SD) Baseline × Post-intervention × 4-week follow-up G1: 26.52 ± 3.24 × 29.65 ± 2.87 × 28.78 ± 2.74 G2: 27.60 ± 2.64 × 29.82 ± 2.08 × 28.95 ± 2.91 *p* < 0.05
Kanyılmaz et al. (2022) [8] Sample: 26 older adults aged ≥65 years (mean age: 70 years) with chronic dizziness VR-Assisted Vestibular Rehabilitation Group (G1): *n* = 13 Conventional Vestibular Rehabilitation Group (G2): *n* = 13	6-month follow-up	BBS score (median [IQR]) Baseline × Post-intervention × 6-month follow-up G1: 50.00 (6.00) × 54.00 (3.00) × 54.00 (4.00) G2: 51.00 (6.00) × 52.00 (5.00) × 51.00 (5.00) *p* = 0.042

Abbreviations: BBS, Berg Balance Scale; G1, Group 1; G2, Group 2; IQR, interquartile range; Mini-BESTest, Mini-Balance Evaluation Systems Test; VR, Virtual reality.

## Data Availability

The data generated by this study will be made available by the corresponding author upon request.

## Supplementary Materials

The following supporting information can be downloaded at https://www.mdpi.com/article/10.3390/geriatrics11050143/s1. S1: PRISMA 2020 for Abstracts Checklist; S2: PRISMA 2020 Checklist; S3: Assessment of the certainty of evidence according to the GRADE Approach [54].

## Abbreviations

The following abbreviations are used in this manuscript:

ADLs	Activities of Daily Living
BBS	Berg Balance Scale
CAVE	Cave Automatic Virtual Environment
FRT	Functional Reach Test
FSST	Four Square Step Test
GRADE	Grading of Recommendations Assessment, Development and Evaluation
LILACS	Literatura Latino-Americana e do Caribe em Ciências da Saúde
LOS	Limits of Stability
MeSH	Medical Subject Headings
MiniBESTest	Mini-Balance Evaluation Systems Test
PEDro	Physiotherapy Evidence Database
POMA	Performance-Oriented Mobility Assessment
PRISMA	Preferred Reporting Items for Systematic Reviews and Meta-Analyses
PROSPERO	International Prospective Register of Systematic Reviews
SciELO	Scientific Electronic Library Online
SLS	Single-Leg Stance
RSRA	Rayyan Systematic Review Accelerator
TLA	Three-letter acronym
TS-EC	Tandem Stance with Eyes Closed
TUG	Timed Up and Go
VR	Virtual Reality
