# Supplementary material for: Effects of Virtual Reality-Based Rehabilitation on Postural Balance in Older Adults: A Systematic Review
Source: Geriatrics (Basel). 2026 Oct 7;11(5):143. doi: 10.3390/geriatrics11050143 (PMC13704171; doi:10.3390/geriatrics11050143)
Supplement: Supplementary file 1 [file geriatrics-11-00143-s001.zip › geriatrics-4500614-supplementary.pdf]

**Material Suplementar 1.** PRISMA 2020 for Abstracts Checklist

| Section and Topic | It | Checklist item | Reported (Yes/No) |
|---|---|---|---|
| **TITLE** | | | |
| Title | 1 | Identify the report as a systematic review. | Page 1 |
| **BACKGROUND** | | | |
| Objectives | 2 | Provide an explicit statement of the main objective(s) or question(s) the review addresses. | Page 1 |
| **METHODS** | | | |
| Eligibility criteria | 3 | Specify the inclusion and exclusion criteria for the review. | Page 1 |
| Information sources | 4 | Specify the information sources (e.g. databases, registers) used to identify studies and the date when each was last searched. | Page 1 |
| Risk of bias | 5 | Specify the methods used to assess risk of bias in the included studies. | Page 1 |
| Synthesis of results | 6 | Specify the methods used to present and synthesise results. | Page 1 |
| **RESULTS** | | | |
| Included studies | 7 | Give the total number of included studies and participants and summarise relevant characteristics of studies. | Page 1 |
| Synthesis of results | 8 | Present results for main outcomes, preferably indicating the number of included studies and participants for each. If meta-analysis was done, report the summary estimate and confidence/credible interval. If comparing groups, indicate the direction of the effect (i.e. which group is favoured). | Page 1-2 |
| **DISCUSSION** | | | |
| Limitations of evidence | 9 | Provide a brief summary of the limitations of the evidence included in the review (e.g. study risk of bias, inconsistency and imprecision). | Not applicable |
| Interpretation | 10 | Provide a general interpretation of the results and important implications. | Not applicable |
| **OTHER** | | | |
| Funding | 11 | Specify the primary source of funding for the review. | Not applicable |
| Registration | 12 | Provide the register name and registration number. | Page 1 |

**Material Suplementar 2.** PRISMA 2020 Checklist

| Section and Topic | Item # | Checklist item | Location where item is reported |
|---|---|---|---|
| **TITLE** | | | |
| Title | 1 | Identify the report as a systematic review. | Page 1 |
| **ABSTRACT** | | | |
| Abstract | 2 | See the PRISMA 2020 for Abstracts checklist. | Page 1-2 |
| **INTRODUCTION** | | | |
| Rationale | 3 | Describe the rationale for the review in the context of existing knowledge. | Page 2 |
| Objectives | 4 | Provide an explicit statement of the objective(s) or question(s) the review addresses. | Page 3 |
| **METHODS** | | | |
| Eligibility criteria | 5 | Specify the inclusion and exclusion criteria for the review and how studies were grouped for the syntheses. | Page 4 |
| Information sources | 6 | Specify all databases, registers, websites, organisations, reference lists and other sources searched or consulted to identify studies. Specify the date when each source was last searched or consulted. | Page 5 |
| Search strategy | 7 | Present the full search strategies for all databases, registers and websites, including any filters and limits used. | Page 5<br>Table 1 |
| Selection process | 8 | Specify the methods used to decide whether a study met the inclusion criteria of the review, including how many reviewers screened each record and each report retrieved, whether they worked independently, and if applicable, details of automation tools used in the process. | Page 6 |
| Data collection process | 9 | Specify the methods used to collect data from reports, including how many reviewers collected data from each report, whether they worked independently, any processes for obtaining or confirming data from study investigators, and if applicable, details of automation tools used in the process. | Page 6 |
| Data items | 10a | List and define all outcomes for which data were sought. Specify whether all results that were compatible with each outcome domain in each study were sought (e.g. for all measures, time points, analyses), and if not, the methods used to decide which results to collect. | Page 6 |
| | 10b | List and define all other variables for which data were sought (e.g. participant and intervention characteristics, funding sources). Describe any assumptions made about any missing or unclear information. | Page 6 |
| Study risk of bias assessment | 11 | Specify the methods used to assess risk of bias in the included studies, including details of the tool(s) used, how many reviewers assessed each study and whether they worked independently, and if applicable, details of automation tools used in the process. | Page 6 |
| Effect measures | 12 | Specify for each outcome the effect measure(s) (e.g. risk ratio, mean difference) used in the synthesis or presentation of results. | Not applicable |
| Synthesis methods | 13a | Describe the processes used to decide which studies were eligible for each synthesis (e.g. tabulating the study intervention characteristics and comparing against the planned groups for each synthesis (item #5)). | Page 6 |
| | 13b | Describe any methods required to prepare the data for presentation or synthesis, such as handling of missing summary statistics, or data conversions. | Not applicable |
| | 13c | Describe any methods used to tabulate or visually display results of individual studies and syntheses. | Page 6 |
| | 13d | Describe any methods used to synthesize results and provide a rationale for the choice(s). If meta-analysis was performed, describe the model(s), method(s) to identify the presence and extent of statistical heterogeneity, and software package(s) used. | Page 6 |

| Section and Topic | Item # | Checklist item | Location where item is reported |
|---|---|---|---|
| | 13e | Describe any methods used to explore possible causes of heterogeneity among study results (e.g. subgroup analysis, meta-regression). | Not applicable |
| | 13f | Describe any sensitivity analyses conducted to assess robustness of the synthesized results. | Not applicable |
| Reporting bias assessment | 14 | Describe any methods used to assess risk of bias due to missing results in a synthesis (arising from reporting biases). | Page 6 |
| Certainty assessment | 15 | Describe any methods used to assess certainty (or confidence) in the body of evidence for an outcome. | Page 7 |
| **RESULTS** | | | |
| Study selection | 16a | Describe the results of the search and selection process, from the number of records identified in the search to the number of studies included in the review, ideally using a flow diagram. | Page 7<br>Figure 1 |
| | 16b | Cite studies that might appear to meet the inclusion criteria, but which were excluded, and explain why they were excluded. | Page 7<br>Figure 1. |
| Study characteristics | 17 | Cite each included study and present its characteristics. | Page 7-8 |
| PRisk of bias in studies | 18 | Present assessments of risk of bias for each included study. | Page 8<br>Table 2. |
| Results of individual studies | 19 | For all outcomes, present, for each study: (a) summary statistics for each group (where appropriate) and (b) an effect estimate and its precision (e.g. confidence/credible interval), ideally using structured tables or plots. | Page 10<br>Table 3. |
| Results of syntheses | 20a | For each synthesis, briefly summarise the characteristics and risk of bias among contributing studies. | Page 8<br>Page 9 |
| | 20b | Present results of all statistical syntheses conducted. If meta-analysis was done, present for each the summary estimate and its precision (e.g. confidence/credible interval) and measures of statistical heterogeneity. If comparing groups, describe the direction of the effect. | Not applicable |
| | 20c | Present results of all investigations of possible causes of heterogeneity among study results. | Page 10<br>Table 3. |
| | 20d | Present results of all sensitivity analyses conducted to assess the robustness of the synthesized results. | Not applicable |
| Reporting biases | 21 | Present assessments of risk of bias due to missing results (arising from reporting biases) for each synthesis assessed. | Page 8 |
| Certainty of evidence | 22 | Present assessments of certainty (or confidence) in the body of evidence for each outcome assessed. | Page 8-9 |
| **DISCUSSION** | | | |
| Discussion | 23a | Provide a general interpretation of the results in the context of other evidence. | Page 15 |
| | 23b | Discuss any limitations of the evidence included in the review. | Page 18 |
| | 23c | Discuss any limitations of the review processes used. | Page 18 |
| | 23d | Discuss implications of the results for practice, policy, and future research. | Page 18 |

| Section and Topic | Item # | Checklist item | Location where item is reported |
|---|---|---|---|
| **OTHER INFORMATION** | | | |
| Registration and protocol | 24a | Provide registration information for the review, including register name and registration number, or state that the review was not registered. | Page 3 |
| | 24b | Indicate where the review protocol can be accessed, or state that a protocol was not prepared. | Page 3 |
| | 24c | Describe and explain any amendments to information provided at registration or in the protocol. | Not applicable |
| Support | 25 | Describe sources of financial or non-financial support for the review, and the role of the funders or sponsors in the review. | Page 19 |
| Competing interests | 26 | Declare any competing interests of review authors. | Page 20 |
| Availability of data, code and other materials | 27 | Report which of the following are publicly available and where they can be found: template data collection forms; data extracted from included studies; data used for all analyses; analytic code; any other materials used in the review. | Page 20 |

*From:* Page MJ, McKenzie JE, Bossuyt PM, Boutron I, Hoffmann TC, Mulrow CD, et al. The PRISMA 2020 statement: an updated guideline for reporting systematic reviews. BMJ 2021;372:n71. doi: 10.1136/bmj.n71. This work is licensed under CC BY 4.0. To view a copy of this license, visit https://creativecommons.org/licenses/by/4.0/

**Supplementary Material 3. Assessment of the Certainty of Evidence According to the GRADE Approach**

| Study | Primary outcome | Study design | Risk of bias | Inconsistency | Indirectness | Imprecision | Publication Bias | GRADE Certainty of Evidence | Justification |
|---|---|---|---|---|---|---|---|---|---|
| Fu et al., 2015 [36] | Risk of falls in frail institutionalized older adults | Single-blind randomized controlled trial (RCT) | Not serious | Not serious | Not serious | Moderate (*n* = 60) | Undetected | ⊕⊕⊕◯ Moderate | PEDro 5/10: randomization and baseline comparability met; blinding of participants/therapists not feasible. Imprecision: total n = 60 (30 per group), below optimal information size for continuous outcomes; between-group CIs not reported. Publication bias: no evidence of selective reporting; trial not registered in searched databases. |
| Yeşilyaprak et al., 2016 [37] | Balance and fall risk (BBS, TUG). | RCT | Moderate (small sample size and potential lack of blinding) | Moderate (similar results across groups) | Low | High, small sample (n=18) | Undetected | ⊕⊕◯◯ Low | PEDro 5/10: small sample and lack of blinding downgrade risk of bias. Inconsistency: similar direction of effects but high variability in baseline BBS scores across groups. Imprecision: total n = 18 (7 vs. 11); very small sample with wide CIs for TUG and BBS changes. |
| Bacha et al., 2018 [30] | Balance, functional mobility, and fall risk. | Clinical trial (likely non-randomized with limited control). | Moderate (potential lack of blinding and unclear randomizatio) | Low | Low | Moderate (small sample) | Undetected | ⊕⊕⊕◯ Moderate | PEDro 5/10: potential lack of blinding and unclear randomization downgrade risk of bias. Imprecision: n = 46 (23 per group); between-group comparison for Mini-BESTest showed significant difference but CIs not reported. |
| Sápi et al., 2019 [38] | Postural control and functional balance tests. | Controlled study (allocation not clearly randomized) | Moderate to high (lack of blinding and potential selection bias) | Moderate | Low | Moderate (unequal sample sizes between groups) | Undetected | ⊕⊕◯◯ Low | PEDro 5/10: lack of blinding and potential selection bias (allocation not clearly randomized) downgrade risk of bias. Inconsistency: mixed results across LOS subdomains. Imprecision: n = 75 but unequally distributed (30 vs. 23 vs. 22); some between-group comparisons underpowered. |
| Sadeghi et al., 2021 [39] | Balance and functional mobility. | RCT | Low (randomization and a structured study protocol) | Low | Low | Moderate (n=64, but divided into 4 groups) | Undetected | ⊕⊕⊕⊕ High | PEDro 6/10: randomization and structured protocol adequate; blinding not feasible but otherwise low risk. Imprecision: total n = 58 divided into 4 groups (~14–15 per group), reducing power for pairwise between-group comparisons; however, consistent significant between-group differences observed. |
| Rebêlo et al., 2021 [40] | Functional balance and fall risk in older adults with balance | RCT with allocation concealment and blinded | Not serious | Not serious | Do not record | Severe (n=37) | Undetected | ⊕⊕⊕◯ Moderate | PEDro 7/10: strong methodological quality. Imprecision: n = 37 (20 vs. 17); small sample with wide CIs for TUG and FRT mean changes; study not powered to detect small between-group |

| | | | | | | | | | |
|---|---|---|---|---|---|---|---|---|---|
| | impairments. | outcome assessor. | | | | | | | differences. |
| Zak et al., 2022 [7] | Functional performance, static balance, gait, and fall risk following virtual reality–based physical therapy programs. | RCT | Serious: baseline differences between groups in POMA-G and balance measures, simple randomization, and participant blinding was unlikely. | Not serious | Not serious | Serious: only 15 participants per group; short follow-up (3 weeks) | Undetected | ⊕⊕⊕◯ Moderate | PEDro 5/10: baseline imbalances and lack of participant blinding downgrade risk of bias. Imprecision: n = 60 but divided into 4 groups (15 each); very small per-group sample for multiple comparisons; 3-week intervention limits generalizability of effect estimates. |
| Kanyılmaz et al., 2022 [8] | Dizziness, balance, functional mobility, and fear of falling in older adults with vestibular disorders. | Single-blind RCT. | Not serious | Not serious | Not serious | Severe (n=32; final analysis n=26) | Undetected | ⊕⊕◯◯ Low | PEDro 6/10: single-blind design acceptable. Imprecision: n = 26 (13 per group) with 6 dropouts in final analysis; small sample limits precision of between-group BBS and TUG differences at 6-month follow-up. |
| Stanmore et al., 2024 [41] | Feasibility, acceptability, and usability of exergaming for fall prevention (recruitment, retention, and System Usability Scale [SUS]). | Cluster-randomized controlled trial (2 institutions; *n* = 24). | Serious: only two clusters, no participant blinding, supervised intervention with potential performance bias, and the study was not designed to evaluate efficacy. | Not serious | Not serious | Small sample (24 participants), lacking statistical power to detect clinical differences. | Suspected: single pilot study with external funding related to the evaluated technology. | ⊕⊕◯◯ Low | PEDro 5/10: cluster design with only 2 clusters and lack of blinding downgrade risk of bias. Imprecision: *n* = 22 (10 vs. 12); pilot study not powered for efficacy; mean difference for BBS (−4.3; 95% CI: −10.7 to 2.1) crosses zero, indicating imprecision. Publication bias: suspected due to pilot nature and technology-related funding source. |

Abbreviations: BBS, Berg Balance Scale; RCT, Randomized controlled trial; POMA-G, Performance-Oriented Mobility Assessment—Gait Subscale; VR, virtual reality; SUS, Brazilian Unified Health System; TUG, Timed Up and Go.

The certainty of evidence for each outcome was evaluated independently by two reviewers using the GRADE framework. The following criteria were applied to each domain. Risk of bias: was judged based on the PEDro scale results, with downgrading applied for lack of allocation concealment, participant/therapist blinding, or attrition. Inconsistency: was assessed by comparing the direction and magnitude of effects across included studies; downgrading occurred when effect estimates varied widely across similar interventions or populations. Indirectness: was evaluated by examining the applicability of the study populations, interventions, comparators, and outcomes to the review question. Imprecision: was assessed qualitatively because a meta-analysis was not performed; studies with fewer than 30 participants per group or wide, uninformative confidence intervals were downgraded for serious imprecision. Publication bias: was not evaluated using funnel plots or Egger's regression due to the limited number of included studies (n < 10); instead, a qualitative assessment was conducted by searching trial registries (ClinicalTrials.gov, PROSPERO) for unpublished or ongoing trials, examining the range of reported effect sizes, and considering study sponsorships. GRADE Certainty of Evidence: ⊕⊕⊕⊕ = High certainty; ⊕⊕⊕◯ = Moderate certainty; ⊕⊕◯◯ = Low certainty; ⊕◯◯◯ = Very low certainty.
